# Resveratrol Induced Premature Senescence Is Associated with DNA Damage Mediated SIRT1 and SIRT2 Down-Regulation

**DOI:** 10.1371/journal.pone.0124837

**Published:** 2015-04-29

**Authors:** Mehtap Kilic Eren, Ayten Kilincli, Özkan Eren

**Affiliations:** 1 Department of Medical Biology, Adnan Menderes University Medical School, Aydın, Turkey; 2 ADU-BILTEM (Adnan Menderes University, Science and Technology Research and Application Center), Aydin, Turkey; 3 Department of Biology, Adnan Menderes University, Aydin, Turkey; German Cancer Research Center, GERMANY

## Abstract

The natural polyphenolic compound resveratrol (3,4,5-trihydroxy-trans-stilbene) has broad spectrum health beneficial activities including antioxidant, anti-inflammatory, anti-aging, anti-cancer, cardioprotective, and neuroprotective effects. Remarkably, resveratrol also induces apoptosis and cellular senescence in primary and cancer cells. Resveratrol’s anti-aging effects both *in vitro* and *in vivo* attributed to activation of a (NAD)-dependent histone deacetylase family member sirtuin-1 (SIRT1) protein. In mammals seven members (SIRT1-7) of sirtuin family have been identified. Among those, SIRT1 is the most extensively studied with perceptive effects on mammalian physiology and suppression of the diseases of aging. Yet no data has specified the role of sirtuins, under conditions where resveratrol treatment induces senescence. Current study was undertaken to investigate the effects of resveratrol in human primary dermal fibroblasts (BJ) and to clarify the role of sirtuin family members in particular SIRT1 and SIRT2 that are known to be involved in cellular stress responses and cell cycle, respectively. Here, we show that resveratrol decreases proliferation of BJ cells in a time and dose dependent manner. In addition the increase in senescence associated β-galactosidase (SA-β-gal) activity and methylated H3K9-me indicate the induction of premature senescence. A significant increase in phosphorylation of γ-H2AX, a surrogate of DNA double strand breaks, as well as in levels of p53, p21^CIP1^ and p16^INK4A^ is also detected. Interestingly, at concentrations where resveratrol induced premature senescence we show a significant decrease in SIRT1 and SIRT2 levels by Western Blot and quantitative RT-PCR analysis. Conversely inhibition of SIRT1 and SIRT2 via siRNA or sirtinol treatment also induced senescence in BJ fibroblasts associated with increased SA-β-gal activity, γ-H2AX phosphorylation and p53, p21^CIP1^ and p16^INK4A^ levels. Interestingly DNA damaging agent doxorubicin also induced senescence in BJ fibroblasts associated with decreased SIRT1/2 levels. In conclusion our data reveal that resveratrol induced premature senescence is associated with SIRT1 and SIRT2 down regulation in human dermal fibroblasts. Here we suggest that the concomitant decline in SIRT1/2 expression in response to resveratrol treatment may be a cause for induction of senescence, which is most likely mediated by a regulatory mechanism activated by DNA damage response.

## Introduction

Resveratrol (3,4,5-trihydroxy-trans-stilbene) is a natural polyphenolic compound which exerts a number of health preserving effects, including antioxidant, anti-inflammatory, anti-aging, cardioprotective, neuroprotective activities [1]. Different studies in cancer and primary cell lines as well as in animal models have connected resveratrol’s anti-oxidant, anti-inflammatory, and growth-inhibitory activities to the inhibition of proliferation in association with cell cycle arrest, induction of apoptotic cell death or senescence [2–5]. Thus, resveratrol has different activities in regulating multiple cellular events associated with carcinogenesis, and aging. Resveratrol’s anti-aging effects both in vitro and in vivo attributed to activation of a (NAD)-dependent histone deacetylase family member sirtuin-1 (SIRT1) protein, the mammalian homologue of yeast Sir2 (silent information regulator 2) [1,6].

Sirtuins are a class of proteins retaining either histone deacetylase or mono-ribosyltransferase activity and have been implicated in various biological processes including aging, regulation of transcription, apoptosis and stress resistance, as well as energy efficiency and alertness under calorie restriction situations [7,8]. In mammals seven members (SIRT1-7) of sirtuin family have been identified which are localized to different subcellular compartments such as the nucleus (SIRT1, -2, -6, -7), cytoplasm (SIRT1 and SIRT2) and the mitochondria (SIRT3, -4 and -5) [7,8]. Among those, SIRT1 is the most extensively studied within the last years, thus, many of its downstream mediators have been identified with perceptive effects on mammalian physiology and suppression of the diseases of aging, though, current evidence does not support that SIRT1 can increase mammalian longevity [9–12]. On the other hand, there are studies indicating that sirtuins are not always committed to cell survival: under different stress conditions, SIRT1, SIRT2, and SIRT3 can protect the organism by inducing cell senescence or apoptosis [13–16]. Interestingly a recent report has suggested that SIRT1 can counteract cellular senescence in human diploid fibroblasts [17].

Cellular senescence is a sustained anti-proliferative response arresting cell cycle. Depending on the activating signals it can be categorized as replicative and premature senescence [18]. Replicative senescence was first described by Hayflick and Moorhead in 1961 in normal mammalian cells characterized by a finite replicative potential, limiting their lifespan to a certain number of divisions [19], currently known to be induced via signals triggered by telomere shortening [20]. Premature senescence, on the other hand, can be induced in young cells via several other mechanisms such as activation of certain oncogenes (e.g. Ras, Braf), inactivation of tumour supressor gene (e.g. Pten) or mitogenic stimulation, DNA damaging agents, oxidative stress [18]. Senescence induced via activated oncogenes now accepted as a tumour suppressor mechanism acting as an initial barrier and preventing cells progression to cancer [21]. Senescence is characterized by a number of morphological and biochemical changes including flattened morphology, increased senescence associated β-galactosidase activity (SA-β-gal) and increase in tri-methylated lysine 9 of histone H3 (H3K9-3me) reflecting the formation of senescence associated heterochromatin foci (SAHF) [22]. The p16^INK4A^-Rb and p53-p21^CIP1^ pathways are well-known tumour suppressor pathways mediating the induction and maintenance of senescence [23]. In addition, DNA damage response also known as crucial mediator of cellular senescence that involves activation of ATM/ATR (Ataxia Talengiectasia Mutataed)/(ATM and Rad3 related), kinases and phosphorylation of the histone variant γ-H2AX [21]. Induction of cellular senescence can be exploited for cancer therapy as it arrests cells proliferation and limits the life span whereas its impact on organismal aging is still debated [18,24,25]. Currently, senescent cells drawing more attention due to their multifaceted effects related to cancer prevention or progression as well as their possible impact on aging [20].

Previously it has been demonstrated that low doses of resveratrol can prevent the growth of cancer cells via induction of premature senescence [26–28]. However, resveratrol has been also shown to induce senescence in normal primary cells [29–31]. Interestingly, yet no data has specified the role of sirtuins, in particular the role SIRT1 under conditions where resveratrol induces premature senescence in primary cells. Thus, resveratrol seems to play a dual and somewhat opposite roles which certainly warrants for further investigations. Current study was undertaken to investigate whether resveratrol induces premature senescence in human primary dermal fibroblasts (BJ) and to clarify the role of sirtuin family members SIRT1 and SIRT2 in this context. Here we show that resveratrol treatment decreases BJ cells proliferation in a time and dose dependent manner associated with significantly increased SA-β-gal activity and methylated H3K9-me. We also detected a significant increase in phosphorylation of γ-H2AX and in expressions of p53, p21^CIP1^ and p16INK4A in BJ cells. Interestingly at concentrations where resveratrol induced premature senescence we detected a significant decrease in SIRT1 and SIRT2 levels. Accordingly knock down of SIRT1 and SIRT2 via siRNA or their chemically inhibition via sirtinol also induced senescence in BJ fibroblasts associated with increased SA-β-gal activity, γ-H2AX phosphorylation and increased p53, p21^CIP1^ and p16^INK4A^ levels. Interestingly in BJ fibroblasts doxorubicin-induced senescence is also associated with decreased SIRT1 and SIRT2 levels. Our results demonstrate that resveratrol induced decrease in SIRT1/2 expression may be a cause for induction of senescence which is most likely mediated by a regulatory mechanism activated by DNA damage response.

## Materials and Methods

### Cell Culture

Human new born foreskin fibroblasts (BJ) were obtained from the American Type Culture Collection (ATCC CRL-2522) and were cultured in Dulbecco’s modified Eagle’s medium (Gibco) supplemented with 10% fetal bovine serum (FBS; Biochrom, Germany) and 100 Units/mL penicillin, 100_g/mL streptomycin, 2 mmol/L glutamine incubated in a humidified chamber at 37°C supplemented with 5% CO_2_. In all experiments cells were used within 20–30 population.

### Sirtinol and Doxorubicin treatments

BJ cells either left untreated or treated with 50 and 100 μmol/L of sirtinol (2-[(2-Hydroxynaphthalen-1-ylmethylene)amino]-N-(1-phenethyl)benzamide; Sigma) or 50 or 100 ng/ml of Doxorubicin, were incubated for three and five days, respectively in a humidified chamber at 37°C supplemented with 5% CO_2_. Culture medium was changed with fresh additives at every 72 h. Subsequently cells were stained at day 5 for SA-β-gal activity and γH2AX foci formation and analysed by Western blot for SIRT1, SIRT2, and p53, p21^CIP1^, and p16^INK4A^ expressions.

### RNA interference

BJ cells were transfected with (50 nmol/l) small interfering RNA oligos targeting SIRT1 (‘5- GACACTGTGGCAGATTGTTATTAAT-3’) and SIRT2 (‘5-GCTCATCAACAAGGAGAAA-3’) (Life technologies, Invitrogen) independently and an inverted siRNA was used as negative control (INC). Transfection was performed by using oligofectamine (Invitrogen) according to the manufacturer’s instruction. 48 hours post transfection; cells were harvested and examined by Western blot analysis for SIRT1 and SIRT2 expressions. 72 h post transfection cells were analysed for p53, p21^CIP1^, and p16^INK4A^ expressions and stained for SA-β-gal activity and γ-H2AX foci formation.

### Cell proliferation assay

The WST-1 proliferation assay (Roche Diagnostic GmbH, Mannheim, Germany) was used to determine the cell viability in cells either left untreated or resveratrol treated. Cells were seeded in 96-well plates at a density of 4x10^3^ cells (in 100 μl/well) and treated with resveratrol for 24, 48, and 72 h at a concentration of 5, 10, 25, 50 or 100 μmol/L. Cells were also treated with DMSO (0,01%) alone as solvent control. After 24, 48 and 72 h incubation, WST-1 reagent was added and incubated for 2 h, and then measured at a wavelength of 450 nm with Multiscan JX (Thermo Labsystems, Vantee, Finland). Cell viability was calculated by the following formula: relative cell viability = (average absorbance of treated group—average absorbance of blank)/(average absorbance of control group- average absorbance of blank). Assays were performed in triplicate and repeated three times.

### BrdU incorporation

BrdU incorporation was performed utilizing Cell Proliferation ELISA, BrdU Assay Kit (Roche Applied Science, Indianapolis, IN) according to the manufacturer’s instruction. In brief, 4x10^3^ cells (in 100 μl/well) were cultured in 96-well plates in complete growth media. At indicated time points cells were labelled using 10 μM BrdU and re-incubated overnight at 37°C in a humidified atmosphere. The next day, the culture media was removed; cells were then treated with FixDenat and subsequently incubated with the anti-BrdU-P OD antibody for 90 minutes at room temperature. After the incubation period cells were washed and supplemented with the substrate solution. Accordingly quantification of the reaction product was measured by using a scanning multi-well spectrophotometer (ELISA reader) at an absorbance of 370 nm with a reference wavelength of 492 nm. For comparison, we show for indicated time points the percentage of BrdU-positive cells in control (untreated) or DMSO (solvent control) or resveratrol treated cells.

### Senescence-Associated β-Galactosidase activity

SA-β-gal activity was detected in cells either left untreated (control) or treated with resevratrol as previously described [32] with minor modifications. DMSO was used as solvent coltrol. In brief at the indicated time points, cells were washed with PBS, fixed with 0.5% glutaraldehyde (PBS [pH 7.2]), and washed in PBS (pH 7.2) supplemented with 1 mM MgCl2. Cells were stained in X-gal solution (1 mg/ml X-gal [Boehringer], 0.12 mM K3Fe[CN]6, 0.12mM K4Fe[CN]6, 1 mM MgCl2 in PBS at pH 6.0) overnight at 37 C. Subsequently all stained cells were photographed with an inverted bright field microscope (Olympos) and digital camera system.

### Tunel Staining

Tunel staining was performed by using *In Situ* Cell Death Detection kit with Fluorescein (Roche Applied Science, Indianapolis, IN) to label apoptotic cells as previously described [33]. Briefly, cells were fixed in 1% paraformaldehyde (PFA) containing Triton X-100 on ice for 45 min. Then pelleted cells were, rinsed, resuspended in ice cold 70% EtOH and stored at -20°C at least overnight to permeabilize prior to suspension in the TdT label/TdT enzyme mix for 1 hr at 37°C in the dark. Labeled cells were rinsed with PBS, re-fixed in 4% PFA on ice for 20 min, resuspended in PBS. According to DAPI (Invitrogen, Carlsbad, CA) staining samples were visualized by fluorescence microscopy using 20X magnification (Olympus). The percentage of TUNEL positive cells was determined using the standard formula for the apoptotic index (AI), which was calculated as follows: AI = (number of TUNEL-positive cells/total number of cells) x 100

### Immunofluorescence analysis

Cells were grown for on cover slips and either left untreated or treated with resveratrol or DMSO (0,01%). At the indicated time points cells were fixed in 4% PFA-PBS and used for immunostaining. First cells were permeabilized with 1% TritonX-PBS for 10 minutes and washed with PBS, subsequent incubation with primary antibodies Ki-67 (clone TEC-3, M-7249, Dako), H3K9me3 and Casp3p17 (07–523, Millipore) and γH2AX (05–636, Millipore) was performed overnight, at 4°C. Next day, cover slips containing cells were then washed and incubated for one hour at 37°C in the dark with the following secondary antibodies (AlexaFluor488 goat anti-rat, AlexaFluor488 goat anti-rabbit and AlexaFluor488 rabbit anti-mouse, (Invitrogen) diluted in 3% BSA in PBS/0.05% Tween. Slides were then washed and counterstained with DAPI for nuclear staining mounted and analysed with fluorescence microscope (Olympos) and photographs were taken by an attached digital camera system. The percentage of Ki-67 positive cells was calculated as: = (number of Ki67 positive cells) / (total number of cells in a field) X100.

### Western Blot Analysis

After indicated time points cells were collected and washed with PBS and lysed in NP-40 lysis buffer (150mM NaCl, 1.0% NP-40, 50 mM Tris–HCl [pH 8.0], 1 mM phenyl- methylsulfonyl fluoride, 1 mg/ml leupeptin, 1 mM sodium vanadate,1mM EDTA) on ice for 20 minutes. Lysates were then cleared by centrifugation, at 13,000 rpm for 10 minutes. 25–50 μg of protein samples were separated on SDS–PAGE gels and transferred to Immobilon-P membranes (Millipore). Membranes were then incubated in primary antibodies against, p53, p21^CIP1^, p16^INK4A^, Caspase-3 (Santa Cruz Biotechnology, San Diego, CA, USA) and SIRT1, SIRT2 and β-actin (Sigma) overnight at 4°C) HRP-conjugated rabbit/mouse secondary antibodies were purchased from (Santa Cruz Biotechnology).Western blot analysis was accomplished according to standard procedures using ECL reagent (Millipore).

### Quantitative Real Time PCR

Total cellular RNA was isolated by using Qiagen Rneasy Mini kits (Qiagen Inc., Valencia, CA, USA). For single-stranded cDNA synthesis, cDNA Synthesis Kit SuperScript III RT (Invitrogen Life Technologies, Carlsbad) and 0,5 μg of total RNA was used. Gene-primers for SIRT1 and SIRT2 were purchased from Applied Biosystems (TaqMan gene expression assay). Quantitative real time PCR (RT-qPCR) was performed with Step One Real Time PCR (Applied Biosystems, Foster City, CA, USA) instrument. Results were normalized using Human β-actin Pre-developed TaqMan assay reagents (Applied Biosystems). Changes in the target mRNA content was determined using a comparative CT method (ABI User Bulletin number 2).

### Microscopy analysis

For senescence associated β-galactosidase (SA-β-gal) detection microscopy analysis was performed with the inverted bright field microscope (Olympus). Fluorescence signals were detected by fluorescence microscopy (Olympus).

For statistical analysis *student’s t test* was performed.

## Results

### Resveratrol decreases BJ fibroblast’s proliferation in a time- and dose-dependent manner

**Fig 1:**
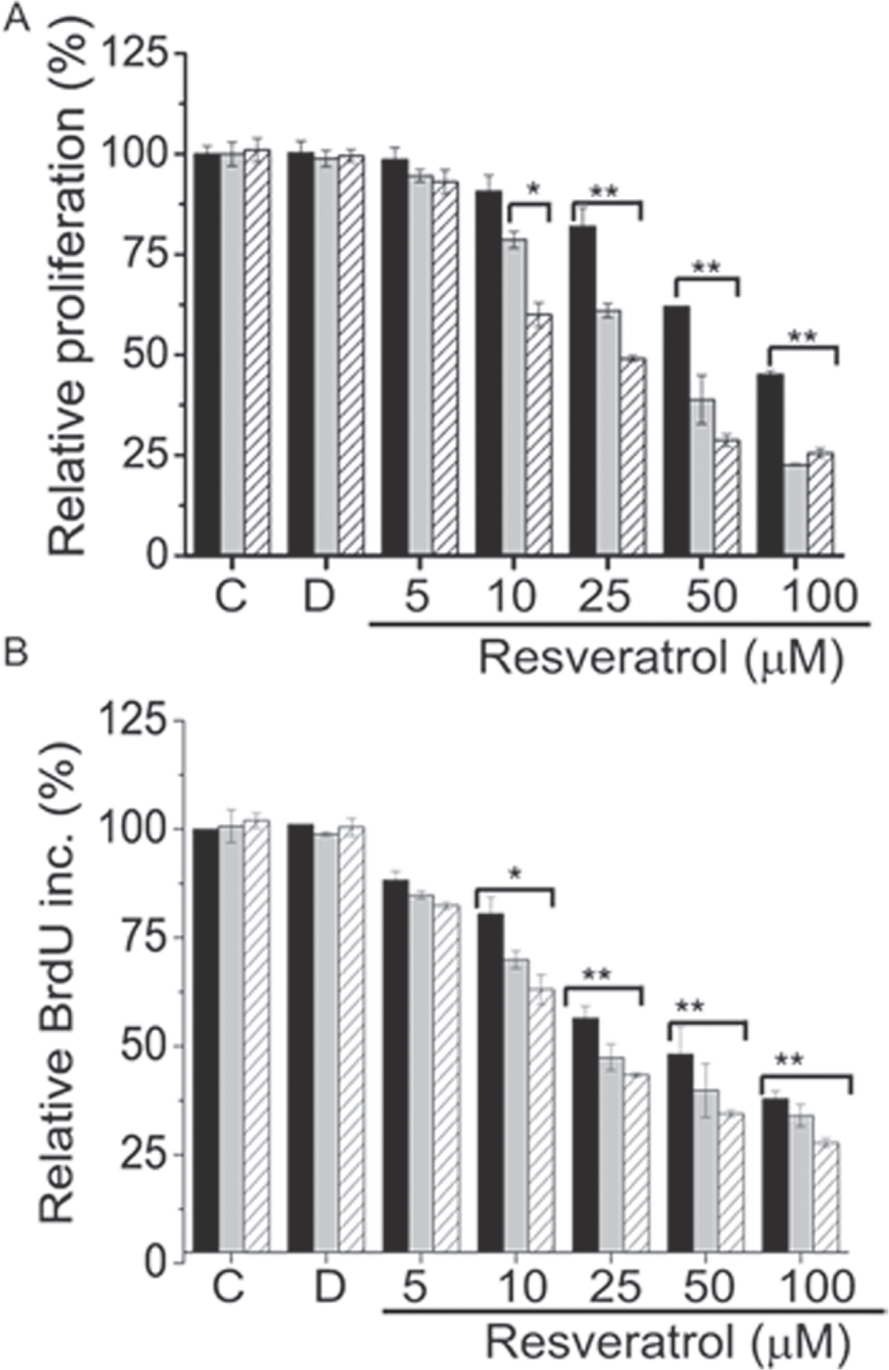
Resveratrol decreases cell proliferation in a time and dose dependent manner. BJ fibroblasts were either left untreated, **C** control, or treated with **D,** DMSO or 5, 10, 25, 50 100 μM of Resveratrol for 24 h (black bars) 48h (grey bars) and 72h (dashed lines) and proliferation was measured by (A) Wst-1 assay. Relative proliferation was calculated by normalization of data from Wst-1 assay to values corresponding to untreated (control) cells and was expressed as % Relative Proliferation. (B) BrdU incorporation into cellular DNA. Relative BrdU incorporation was calculated by normalization of data to values corresponding to untreated (control) cells and are expressed as % Brdu incorporation. Mean ± s.d. of three independent experiment of is shown * represents p < 0, 05, ** represents p<0,01 using the Student’s *t*-test.

**Fig 2:**
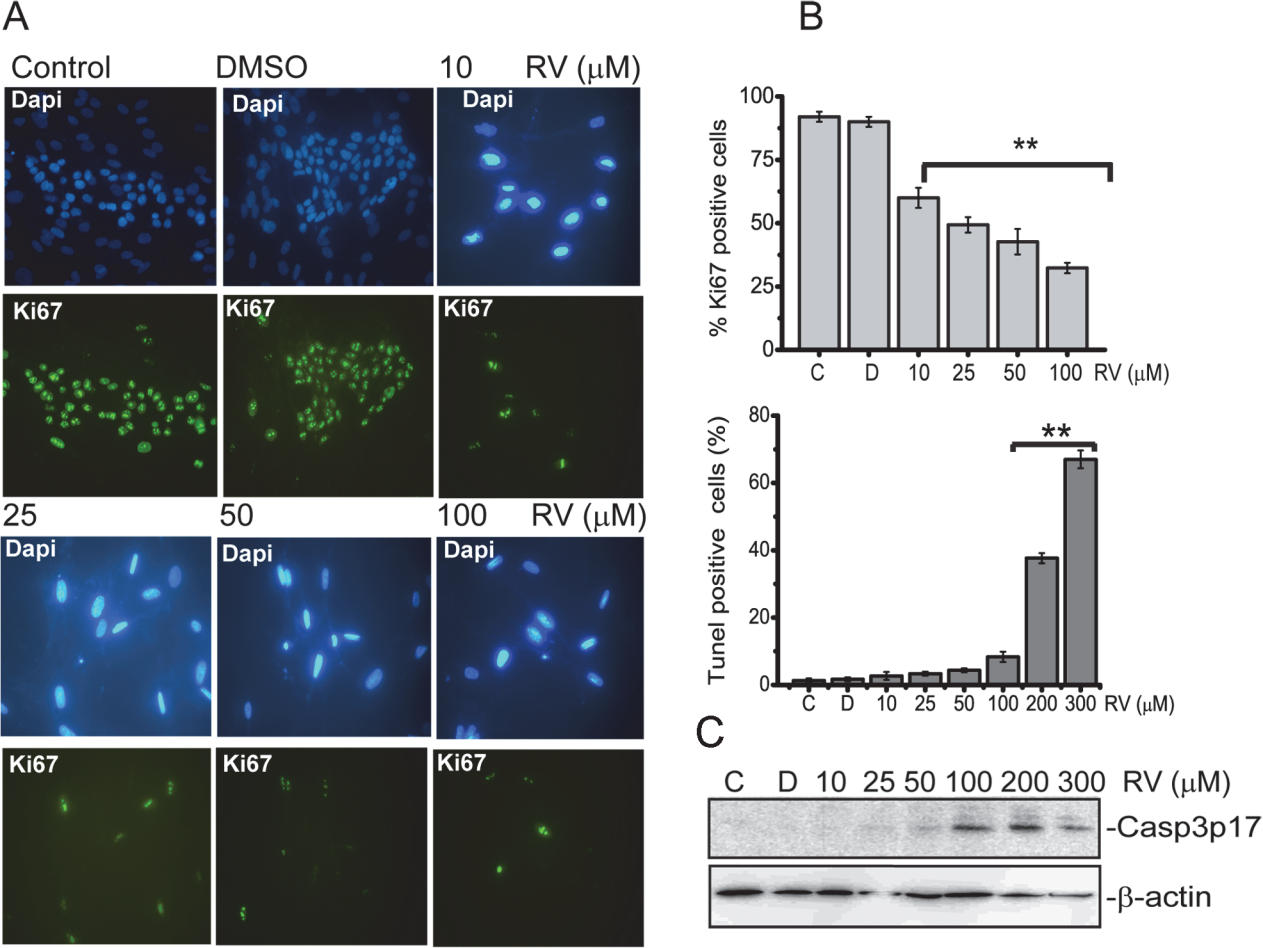
Expression of Ki67 antigen and induction of apoptosis in response to resveratrol treatment. BJ fibroblasts were either left untreated **C** control, or treated with **D,** DMSO or, 10, 25, 50 100 μM of Resveratrol for 72 h. (A) Immunofluorescence staining of Ki67. DAPI was used to counterstain nuclei. Bar graphs show percentage of Ki67-positive cells. BJ fibroblasts were either left untreated **C** control, or treated with **D,** DMSO or, 10, 25, 50 100, 200, 300 μM of Resveratrol for 72 h and analysed (B) for apoptosis by TUNEL staining. Bar graphs show percentage of Tunel positive *cells*. (C) for Western blotting analysis of cleaved caspase-3expression. β-actin was used as loading control. For Ki67 and Tunel stainings the data represent the average and standard deviation of three independent counts of 100 cells each. Mean ± s.d. of three independent experiment of is shown ** represents p<0,01 using the Student’s *t*-test.

Initially to determine the effects of resveratrol on proliferation of BJ cells we performed a time and concentration-response analysis via Wst-1 proliferation assay. Results from Wst-1 assay showed that resveratrol had no significant effect on BJ cells proliferation at a concentration of up to 10 μM during 72h incubation. However starting with 10 μM, increasing concentrations (25, 50, 100 μM) of resveratrol significantly reduced cell proliferation within 24 h incubation, which was further decreased at 48h time point and reached to a maximum level at 72 h time point Fig 1A). Next, in order to confirm data from Wst-1 proliferation assay we engaged BrdU incorporation assay using the same concentrations and time points. As shown in Fig 1B., similar results were obtained from BrdU assay; with increasing concentrations of resveratrol (10, 25, 50, 100 μM), Brdu incorporation in to cellular DNA was gradually decreased during 24h, 48h incubation periods and maximum level of inhibition was detected at 72h, indicating resveratrol had significant inhibitory effect on BJ cell’s proliferation in a time and dose dependent manner. We then assessed proliferation also by detection of the expression of Ki-67 antigen which is a widely used marker for measuring the growth fraction of a given cell population (Fig 2A). Since we measured the maximum inhibition of proliferation at 72h time point we stained for Ki67 expression only at this time point utilizing the same concentrations. Immunofluorescence analysis showed that Ki-67 antigen expression is significantly decreased in BJ cells treated with the increasing concentrations of resveratrol (Fig 2A and 2B). Since we found that resveratrol decreases proliferation and inhibits growth of BJ cells we asked whether apoptosis was induced. Accordingly, we treated cells with same concentrations of resveratrol and measured apoptosis after 72h and found that resveratrol did not induce apoptosis at concentrations of 10, 25, 50μM but starting with 100 μM the percentage of apoptotic cells was increased to 8,3 ±1,5 (Fig 2C). When we increased the concentrations up to 200 and 300 μM, the percentage of apoptotic cells was significantly increased and reached to (37 ±1,5) and (67±2,6) (Fig 2C), respectively. Additionally we measured apoptosis by analysing cleaved Caspase-3 expression under same conditions. As seen in Fig 2C cleaved caspase-3 was detectable in lysates of BJ fibroblasts treated with 100 to 300 μM of resveratrol. Thus, these results clearly show that in BJ fibroblasts resveratrol decreases proliferation in a time and dose-dependent manner and induce apoptosis only at higher concentrations between 100–300 μM.

### Resveratrol induces premature senescence in BJ fibroblasts

**Fig 3:**
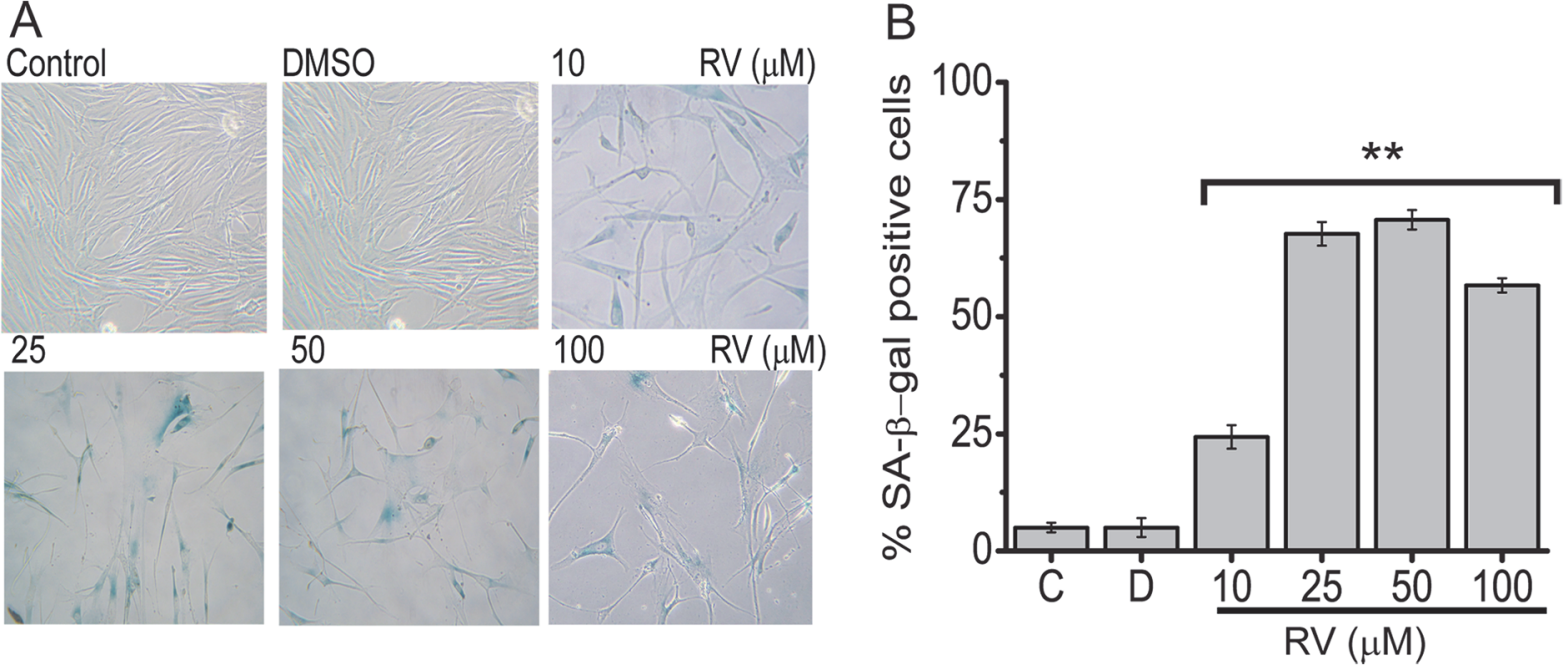
Resveratrol induces premature senescence in BJ fibroblasts. Cells were either left untreated, **C** (control), or treated with **D,** (DMSO) or 10, 25, 50 and 100 μM of Resveratrol for 72 h (A) used for assessment of SA-β-gal activity. SA-b-gal staining increased with RV doses in BJ cells (B) The percentage of SA-β-gal positive senescent cells in RV-treated and control or DMSO treated cells is presented as mean ± s.d of three independent experiments. The data represent the average and standard deviation of three independent counts of 100 cells each. Mean ± s.d. of three independent experiment of is shown, ** represents p<0,01 using the Student’s *t*-test.

Since we found that resveratrol decreases proliferation in BJ cells and apoptosis was not the main response at these concentrations, we investigated whether or not resveratrol treatment induces premature senescence in BJ cells. Increased SA-β-gal activity is a well-known marker of senescence [32], hence we measured senescence via SA-β-gal staining. As shown in “Fig 3A”, the number of SA-β-gal positive senescent cells was significantly increased in resveratrol-treated cells compared to control or DMSO treated cells. Furthermore, the percentage of SA-β-gal positive cells increased with the concentrations of resveratrol indicates that resveratrol induces premature senescence in a dose-dependent manner (Fig 3A and 3B).

**Fig 4:**
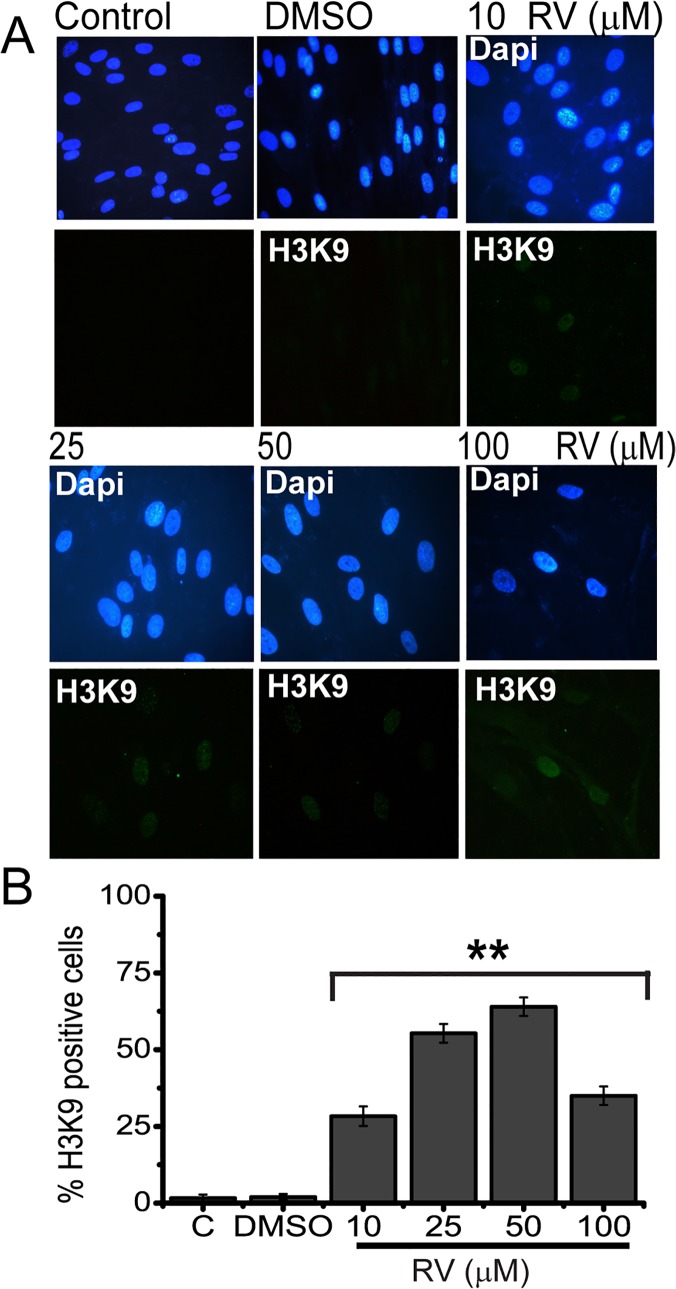
Resveratrol increases H3K9-me in BJ fibroblasts. (A) Immunofluorescence analysis of H3K9-me. Cells were either left untreated, **C** (control), or treated with **D,** (DMSO) or 10, 25, 50 and 100 μM of Resveratrol for 72 h. DAPI was used to counterstain nuclei (B) Quantitation of the percentage of H3K9-me positive cells. The data represent the average and standard deviation of three independent counts of 100 cells each. Mean ± s.d. of three independent experiment of is shown * represents p < 0, 05, ** represents p<0,01 using the Student’s *t*-test.

Accumulation of senescence-associated heterochromatic foci (SAHF) [34], is known as areas of condensed and transcriptionally silenced DNA, and a characteristic of senescence which can be detected by DAPI and H3K9-me3 co-staining. Thus we also tested whether resveratrol treatment results in generation of SAHFs in BJ cells via DAPI and H3K9-me3 co-staining. Results showed that H3K9-me3 positive stained cells were also increased in BJ cells treated with the increasing concentrations of resveratrol (Fig 4A and 4B). These results clearly showed that resveratrol causes premature senescence in BJ fibroblasts.

### Resveratrol induced senescence is mediated by DNA damage and involves activation of p53-p21^CIP1^ and p16INK4A

**Fig 5:**
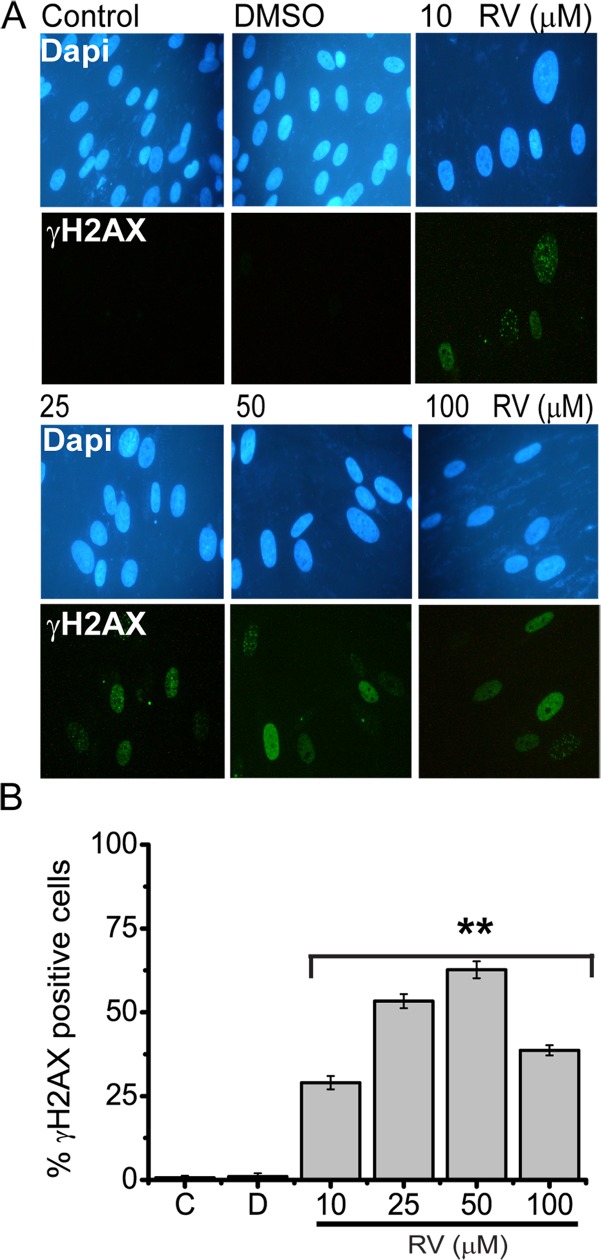
Resveratrol treatment induces formation of γH2AX foci. BJ fibroblasts either left untreated, **C** (control), or treated with **D,** (DMSO) or 5, 10, 25, 50 100 μM of Resveratrol for 72 h and used for (A) Immunofluorescence analysis for γH2AX foci formation DAPI was used to counterstain nuclei (B) Quantification of the number of H2AX foci. Histogram indicates the number of cells containing 5–10 foci. The data represent the mean and ± s.d. of three independent counts of 100 cells each, ** represents p<0,01 using the Student’s *t*-test.

Recent studies have shown that there is a casual link between senescence induction and DNA damage response (DDR). Formation of DNA damage foci containing activated γ -H2A.X (gamma-H2A.X) at either uncapped telomeres or persistent DNA strand breaks is now recognised as an indication for DNA damage and activation of DDR. Consequently, γ-H2A.Xstaining is established as a reliable quantitative indicator of DNA damage response as well as senescence [35]. Accordingly we analysed the levels and activity of DDR by means of γ-H2A.X staining in resveratrol treated cells. As shown in “Fig 5A and 5B” starting with 10 μM resveratrol treatment BJ cells were positively stained for γ-H2A.X and the percentage of positive stained cells were further increased by use of higher concentrations of resveratrol. Taken together these results suggest that resveratrol causes formation of γ-H2A.X foci thus DNA damage which triggers cellular senescence in BJ fibroblasts.

**Fig 6:**
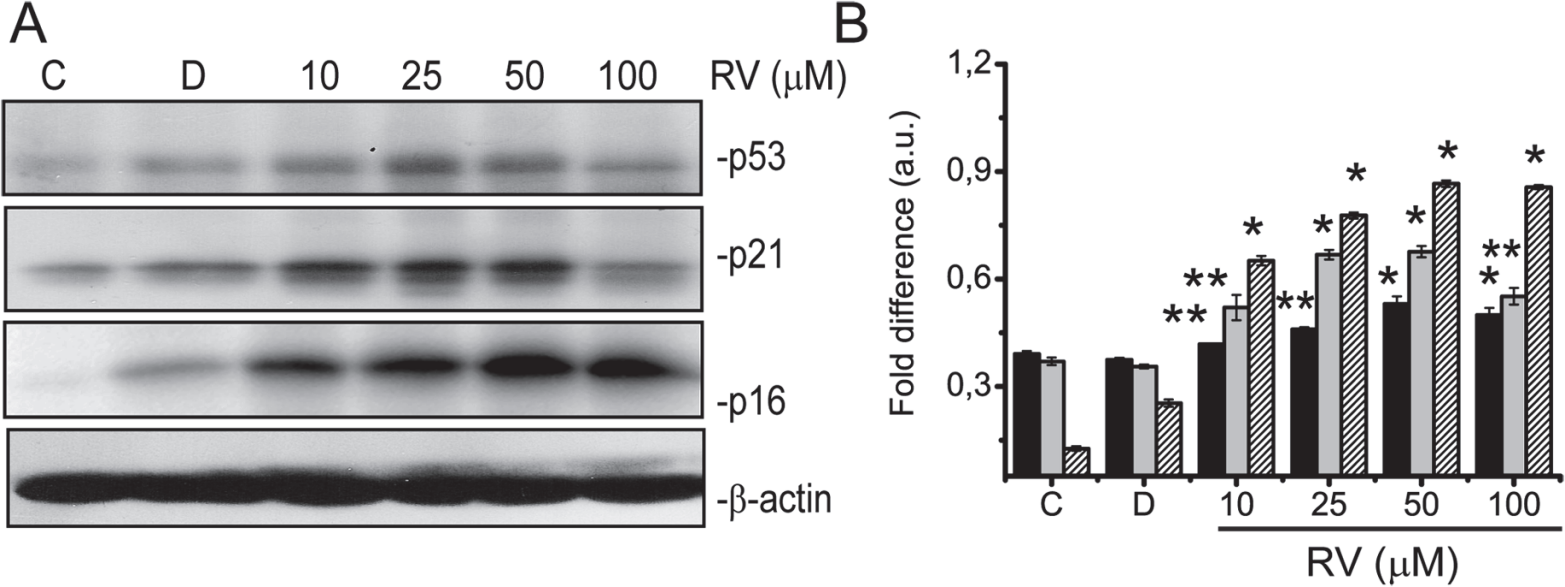
Resveratrol induces senescence through activation of p53, p21^CIP1^ and p16^INK4A^. BJ fibroblasts were either left untreated, **C** (control), or treated with **D,** (DMSO) or 10, 25, 50 and 100 μM of Resveratrol for 72 h and (A) used for Western blotting analysis of p53 and p21 expression. β-actin was used as loading control (B). Western blots obtained as indicated in A. were densitometrically analysed and fold difference expressed as arbitrary units (a.u.). Black bars show p53, grays show p21^**CIP1**^ and dashed bar lines show p16^**INK4A**^ levels. Shown are means ± SD of three independent experiments. * represents p < 0, 05 *vs*. control, ** represents p<0,01 *vs*. control using the Student’s *t*-test.

P53 and p21^CIP1^ and p16^INK4A^ are key molecules involved in the execution of senescence; hence we examined the expression levels of p53, p21^CIP1^ and p16^INK4A^ in resveratrol treated BJ fibroblasts. As shown by Western blotting the expression levels of p53, p21^CIP1^ and p16^INK4A^ were significantly increased upon 10 μM of resveratrol treatment in BJ cells, compared to control or DMSO (Fig 6A and 6B). These data suggest that resveratrol induced premature senescence is mediated by DNA damage and involves activation of p53-p21 pathway as well as activation of p16^INK4A^ in BJ fibroblasts.

### Resveratrol induced senescence is associated with attenuated SIRT1 and SIRT2 expression

**Fig 7:**
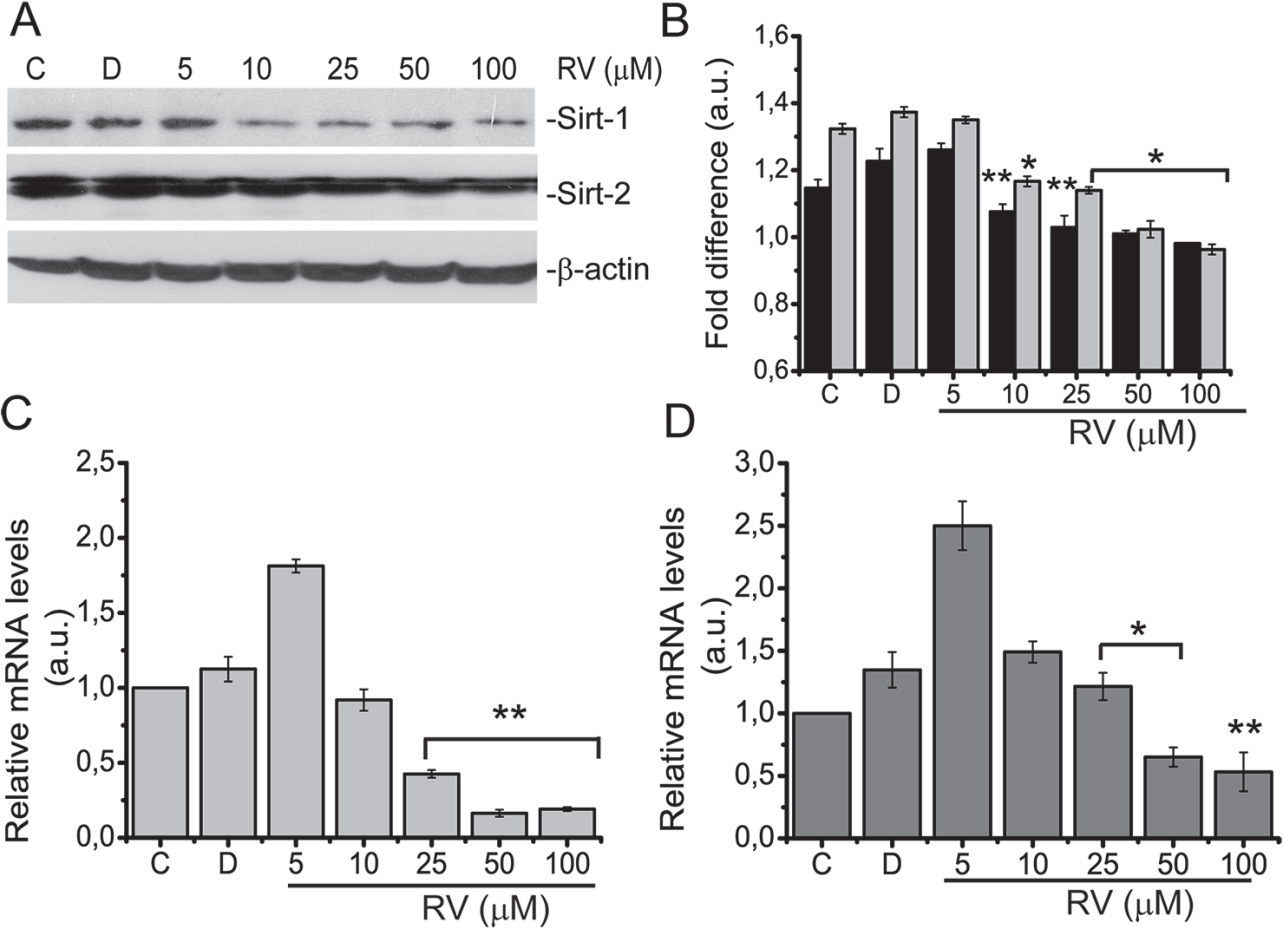
Resveratrol treatment decreases SIRT1 and SIRT2 levels. BJ fibroblasts were either left untreated, **C** (control), or treated with **D,** (DMSO) or 10, 25, 50 and 100 μM of Resveratrol for 72 h and (A) used for Western blotting analysis of SIRT1 and SIRT2 expressions. β-actin was used as loading control (B) Western blots obtained as indicated in A. were densitometrically analysed and fold difference expressed as arbitrary units (a.u.). Black bars show SIRT1 and gray bars show SIRT2 levels. Expression of (C) SIRT1 (histogram with light gray bars) and (D) SIRT2 (histogram with dark gray bars) were analysed for mRNA level by Quantitative RT-PCR. Shown are means ± SD of three independent experiments. * represents p < 0, 05 *vs*. control, ** represents p<0,01 *vs*. control using the Student’s *t*-test.

Previous studies have reported resveratrol, as an activator of Sir2 enzymes in vivo and in vitro. Resveratrol was shown to increase life span in three model organisms through a Sir2-dependent pathway [1]. Furthermore different studies suggest either senescence promoting or preventing role for sirtuins in particular for SIRT1 in different cell types [13,14]. Because we found that resveratrol induce premature senescence in BJ fibroblasts, we speculated whether or not the resveratrol induced senescence was dependent on sirtuins. We analysed expression of SIRT1 and SIRT2 the two members of sirtuin family known to be involved in cellular stress responses and cell cycle, respectively. Interestingly, Western blotting analysis showed that expression of SIRT1 and SIRT2 proteins were significantly decreased upon 10 μM resveratrol treatment and also continued at higher concentrations (25, 50 and 100 μM) (Fig 7A and 7B). We confirmed these data by RT-qPCR analysis and showed that mRNA level of SIRT1 and SIRT2 was also significantly decreased starting with 10μM resveratrol treatment in BJ fibroblasts (Fig 7C and 7D).

### Inhibition of SIRT1 and SIRT2 by siRNA or Sirtinol induces senescence in BJ fibroblasts

**Fig 8:**
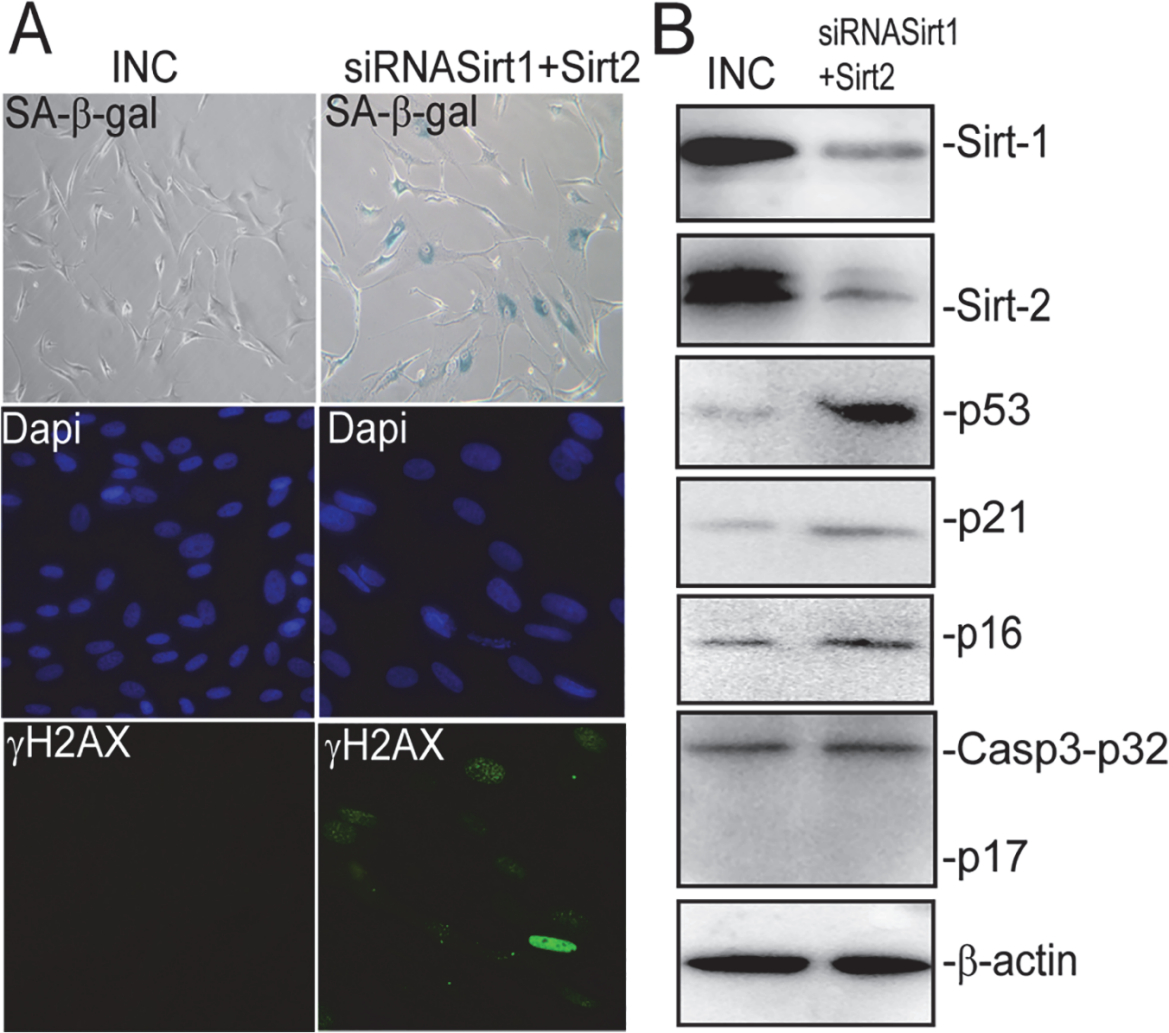
Targeting SIRT1/2 via siRNA induces senescence in BJ fibroblasts. BJ fibroblasts were transfected with siRNA oligos targeting SIRT1/2 or an inverted negative control (**INC**) and 48h post transfection stained for (A) SA-β-galactivity and γ-H2AX foci formation. Dapi was used to counterstain nuclei. (B) analysed for the expression of SIRT1, SIRT2, p53, p21^**CIP1**^, p16^**INK4A**^ and full length (p32) and cleaved (p20) Caspase-3 levels by WB. β-actin was used as loading control.

Next we used RNA interference to knock down SIRT1 and SIRT2 expressions in order to answer the question whether or not down regulation of SIRT1/2 involved in induction of senescence in BJ fibroblasts. Accordingly transfection of specific siRNA oligos independently targeting SIRT1 and SIRT2 significantly decreased expression of SIRT1/2 (Fig 8A) and induced senescence as shown by increased SA-βgal activity in BJ fibroblasts (Fig 8B). Induction of senescence is mediated by DNA damage as evidenced by formation of-H2A.X foci (Fig 8B) and activation of p53-p21^CIP1^ pathway (Fig 8A). A slight increase in levels of p16^INK4A^ was also detected (Fig 8A). Though, apoptosis was not detectable at this time point as we did not detect expression of cleaved caspase-3 (Fig 8B).

**Fig 9:**
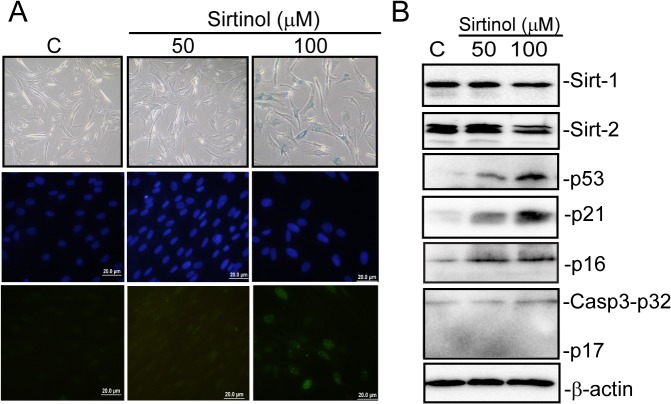
Inhibition of SIRT1/2 by sirtinol induces senescence in BJ fibroblasts. BJ fibroblasts were treated with 50 and 100 μM sirtinol for three days and subsequently (A) analysed for expression of SIRT1, SIRT2, p53, p21^**CIP1**^, p16^**INK4A**^ and and full length (p32) and cleaved (p20) Caspase-3 levels by WB. β-actin was used as loading control (B) stained for SA-β-galactivity and γ-H2AX foci formation. Dapi was used to counterstain nuclei.

Upon finding that genetic knock down of SIRT1 /2 induces senescence we asked whether or not chemical inhibitors of sirtuin family members show similar effects. We used a well-known chemical inhibitor, namely sirtinol in order to repress SIRT1/2 activity as suggested in previous reports [6]. As shown in “Fig 9A” 100 μM sirtinol treatment induced senescence in BJ fibroblasts as evidenced by increased SA-βgal activity (Fig 9A). Consistent with previous reports [36,37] we detected a slight decrease in SIRT1/2 expressions in BJ fibroblasts in response to sirtinol treatment suggesting SIRT1/2 activity might also play a role in regulation of sirtinol induced senescence. Additionally, increased levels of p53, p21^CIP1^ and p16 ^INK4A^ expressions were also detected by sirtinol treatment. More importantly 100 μM of sirtinol induced γ -H2A.X foci formation indicating to the activation of DNA damage response (Fig 9B). However no cleaved caspase-3 expression was detected with 100 μM of sirtinol treatment indicating apoptosis is not induced at this concentration in BJ fibroblasts (Fig 9A).

### Doxorubicin induced senescence is associated with reduced SIRT1 and SIRT2 expressions

**Fig 10:**
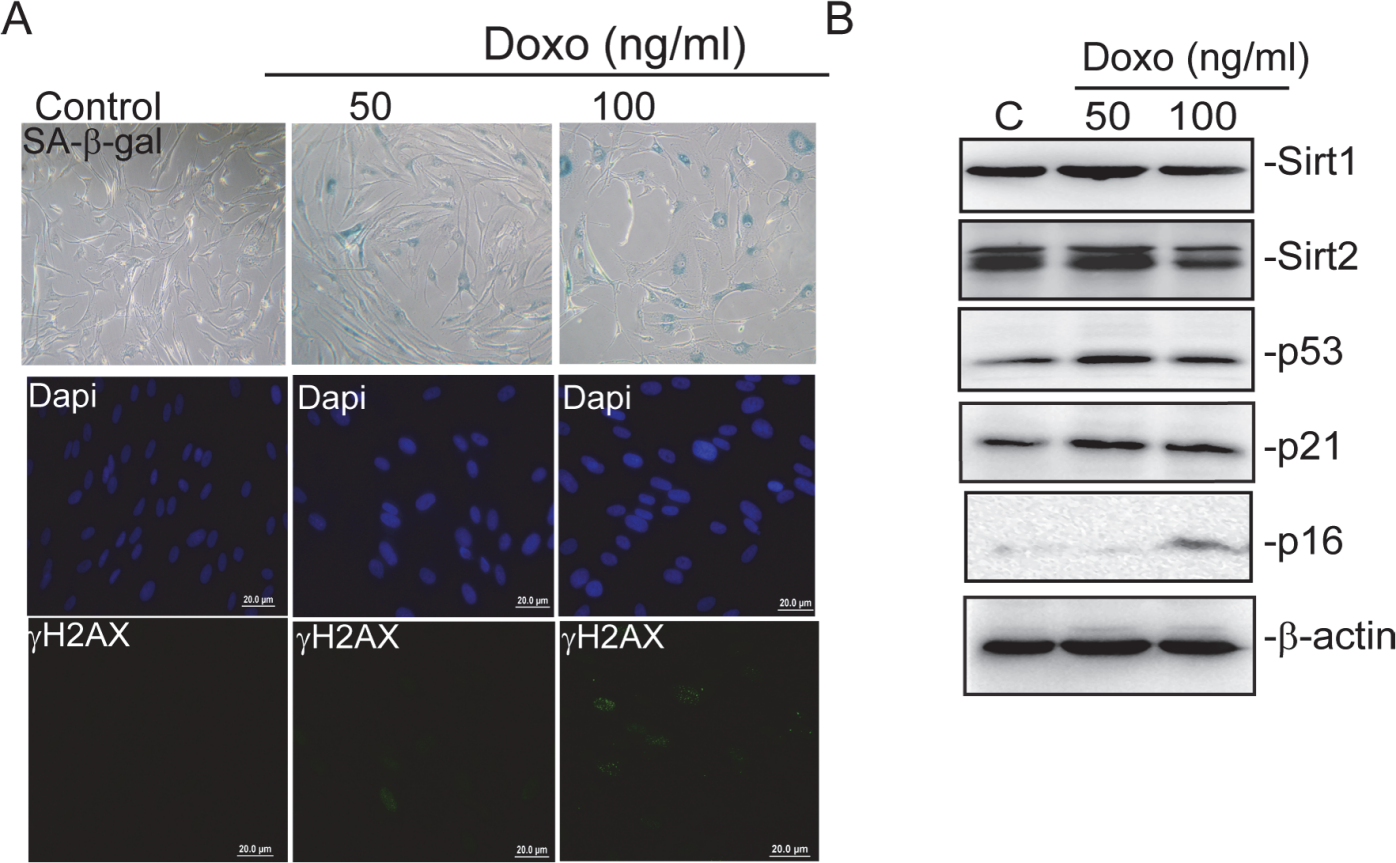
Doxorubicin induced senescence is associated with reduced SIRT1and SIRT2 levels. BJ fibroblasts were treated with 50 and 100 ng/ml of doxorubicin for 5 days and subsequently (A) stained for SA-β-galactivity and γ-H2AX foci formation. Dapi was used to counterstain nuclei. (B) analysed for the expression of SIRT1, SIRT2, p53, p21^**CIP1**^, p16^**INK4A**^ levels by WB. β -actin was used as loading control.

Since we found that resveratrol induced senescence is mediated by DNA damage and down regulation of SIRT1 and SIRT2 expressions we asked whether or not DNA damaging agents that are capable of inducing senescence can reduce expressions of SIRT1/2. Thus in order to induce senescence we treated BJ cells with 50 and 100 ng/ml of doxorubicin for 5 days as suggested in literature [38]. As shown in “Fig 10A”, induction of senescence was evident with increased SA-β-gal activity, increased levels of p53 and p21^CIP1^ and γ-H2A.X foci formation. Additionally, when we tested p16 ^INK4A^ levels we found rather minor increase in p16^INK4A^ levels suggesting doxorubicin induced senescence is mediated mainly by activation of p53-p21 pathway (Fig 10A). Remarkably WB analysis showed that expressions of SIRT1/2 were also slightly reduced during doxorubicin induced senescence (Fig 10B). These data suggest that DNA damage induced senescence is also associated with SIRT1/2 decrease.

Taken together our data clearly show that resveratrol induced senescence is mediated by DNA damage, and mainly by activation of key players of senescence such as p53, p21^CIP1^ and p16^INK4A^. More importantly we show here that there is a concomitant decline in levels of SIRT1 and SIRT2 associated with resveratrol as well as DNA damage induced senescence in BJ fibroblasts suggesting these processes might be governed by a common regulatory mechanism involving DNA damage response.

## Discussion

Resveratrol is a polyphenolic compound with broad spectrum health beneficial activities including antioxidant, anti-inflammatory, anti-aging, anti-cancer, cardioprotective, neuroprotective effects [1]. Resveratrol’s anti-oxidant, anti-inflammatory, and growth-inhibitory effects are mainly attributed to the inhibition of proliferation in association with cell cycle arrest, induction of apoptotic cell death or senescence [1,6]. Resveratrol’s anti-aging effects both in vitro and in vivo attributed to activation of a sirtuin family member SIRT1, though current data does not support that SIRT1 can increase mammalian longevity. On the other hand studies showing SIRT1, SIRT2, and SIRT3 can protect the organism by inducing cell senescence or apoptosis indicated that sirtuins are not always committed to cell survival. Interestingly, controversial reports have shown that SIRT1 counteracts cellular senescence in human diploid fibroblasts [17]. [14,39]. Thus, this study has been conceived to clarify the role of sirtuins during resveratrol induced senescence in BJ fibroblasts. Here, we show that resveratrol decreases proliferation of human dermal fibroblasts in a time and dose dependent manner associated with the induction of premature senescence. Resveratrol induced premature senescence is mediated by DNA damage and involves activation of p53 and p21^CIP1^ and p16^INK4A^. More importantly resveratrol induced senescence is associated with a concomitant decrease in SIRT1 and SIRT2 levels which is most likely mediated by DNA damage in BJ fibroblasts. We provide a number of evidence supporting this conclusion. At first, by employing three different assays including Wst-1, Brdu incorporation and Ki67 staining we showed that starting with 10 μM of resveratrol treatment, proliferation of BJ fibroblast’s decreases in a time- and dose-dependent manner. Importantly at these concentrations apoptosis is not detectable. Accordingly we showed that at same concentrations where proliferation is decreased resveratrol induces premature senescence in BJ fibroblasts as evidenced by senescence hallmarks such as increased SA-β-gal activity, and increased H3K9me3 marks reflecting the formation of SAHFs. Previously Demidenko and Blagosklonny also analysed the effects of resveratrol on human embryonic lung fibroblasts WI-38 and found that resveratrol prevents senescence but this was rather at high, near-toxic concentrations [29]. On the other hand Faragher et al. [30] showed that above 25μM resveratrol concentrations produces a dose dependent reduction in proliferation of human foetal lung fibroblasts associated with increased SA-β-gal activity. In general, our data are in line with these reports; the only slight difference is that in BJ (foreskin) fibroblasts as low as 10 μM of resveratrol can induce senescence whereas 100μM or over induce apoptosis. Hence, we suggest that the differences between resveratrol concentrations may result from the cell types. Recent data has shown that low doses (10–50 μM) of resveratrol induce senescence in lung cancer cells suggesting that resveratrol may exert its anticancer and chemo-preventive effects also through the induction of premature senescence [26]. However, our data showing low concentrations of resveratrol induces senescence in human dermal fibroblasts suggesting that this activity of resveratrol is not selective for cancer cells. More importantly, increasing evidences suggest deleterious effects of senescent cells on the tissue microenvironment due to senescence-associated secretory phenotype (SASP) turning senescent fibroblasts into pro-inflammatory cells which consequently contribute to promote tumour progression [40]. Thus our findings also point out to an important issue for cancer therapy and warrants for further investigations to explore whether or not resveratrol induced SASP may become an important concern for cancer therapy in future.

Recent reports indicate to resveratrol’s DNA damaging effects or its ability to induce senescence involving DNA damage and activation of p53-and p21^CIP1^ pathway [26]. In line with recent reports, in our study we show γ-H2AX foci formation (an essential surrogate of DNA DSBs [35]) during resveratrol induced senescence, suggesting eventually senescence is mediated by DNA damage in BJ fibroblasts. Beside, p53 and p21^CIP1^, p16^INK4A^ levels are also significantly increased suggesting both p53-p21 and Rb-p16 pathways play key role in induction and maintenance of resveratrol induced senescence. More importantly here we show that there is a concomitant decline in mRNA and protein levels of SIRT1 and SIRT2 during resveratrol induced senescence in BJ fibroblasts. We’ve verified this data by showing that SIRT1/2 inhibition either by sirtinol treatment or via RNA interference induces DNA damage mediated senescence in BJ fibroblasts as evidenced by increased SA-βgal activity and increased p53, p21CIP1 and p16INK4A levels. Consistent with our findings a previous report has indicated that the level of SIRT1 also decreases with serial cell passages as cells approach to replicative senescence [25]. Thus, according to literature in mouse and human cells as proliferation decreases either in vivo or in vitro, there is a concomitant decline in the level of SIRT1. In our study in addition to SIRT1 we show that the level of SIRT2 is also decreased as proliferation decreases. More importantly our data which show γH2AX foci formation in response to sirtinol or siRNA treatment suggesting SIRT1/2 inhibition is mediated by DNA damage response. We provide additional data supporting this conclusion by showing that expression of SIRT1/2 were also slightly decreased during DNA damage induced (doxorubicin) senescence. Hence, here we suggest that the concomitant decline in SIRT1/2 levels in response to resveratrol treatment may be a cause for induction of senescence. Interestingly supporting our assumption recent reports suggest, the tumor supresssor gene HIC1 (hypermethylated in Cancer 1) which is widely expressed in healthy tissues but deleted in cancer is activated by DNA double strand breaks and plays a central role in the DNA damage response and DNA repair through the establishment of several complex regulatory loops involving, HIC1, p53, HDAC4, SIRT1 and E2F1[41–43]. Chen and colleagues [42] put forward a model of the HIC1-SIRT1-p53 regulatory loop in which under normal conditions, stress-induced rapid accumulation of p53 activates the HIC1 gene. HIC1 then binds to SIRT1 to form a transcriptional repression complex. The SIRT1/HIC1 complex is recruited to the SIRT1 promoter to suppress SIRT1 transcription. Reduced SIRT1 levels are apparently responsible for increased acetylation level of p53, thereby facilitating its functions of cell cycle arrest, DNA repair and apoptosis [42]. Thus taken together, we cannot rule out the possibility that activated HIC1 might be involved in regulation of SIRT1/2 levels in BJ fibroblasts. To our knowledge our study is the first demonstration of down regulation of both SIRT1 and SIRT2 upon induction of senescence in response to resveratrol or doxorubicin treatment in human primary cells. Currently our knowledge on the mechanism of SIRT1 and SIRT2 down regulation and its contribution to DNA damage induced senescence or *vice versa* is limited, nevertheless, an effort is underway to fully understand the mechanism(s) of down regulation and the relationship with DNA damage response.

**Fig 11:**
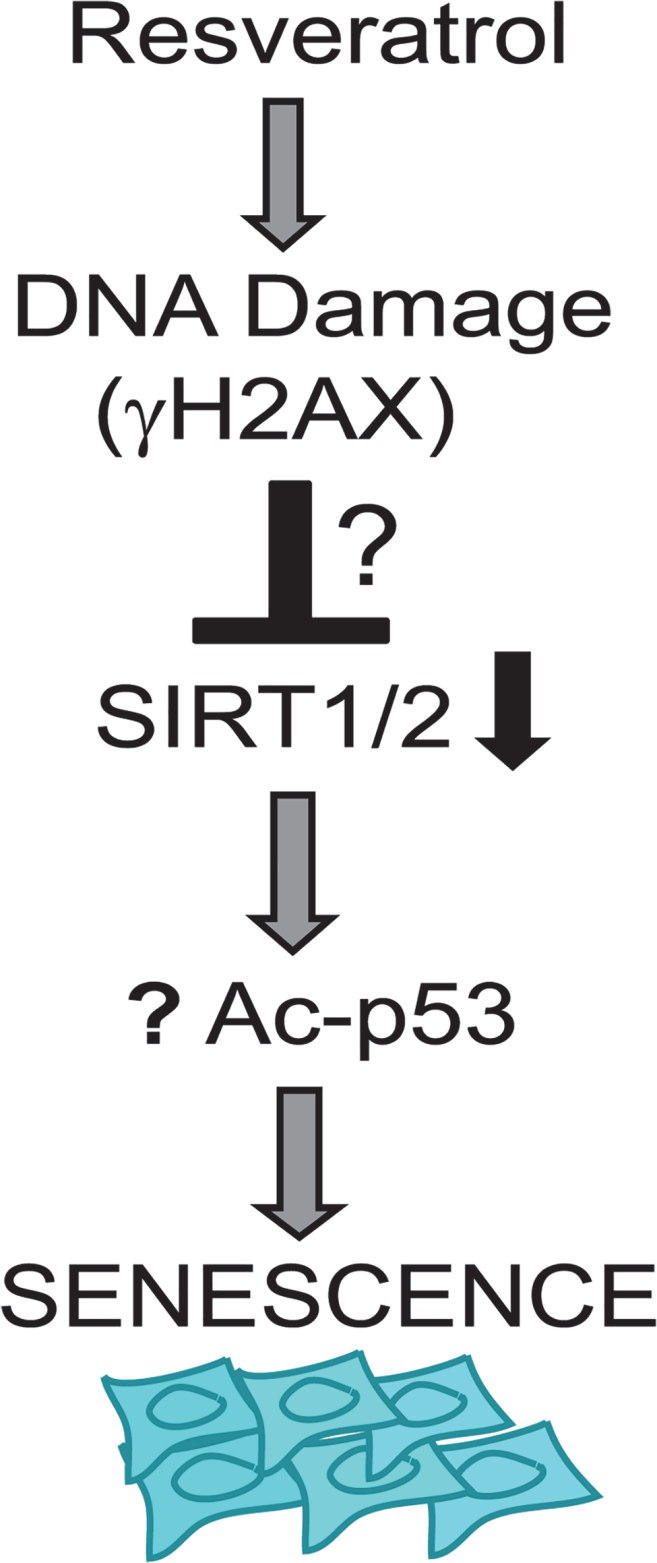
A possible schematic model for mechanism of resveratrol induced premature senescence in BJ fibroblasts. Resveratrol induced senescence is associated with DNA damage, and decreased expression of SIRT1/2. Concomitant decline in the levels of SIRT1 and SIRT2 upon resveratrol treatment may be responsible for increased acetylation level of p53, which in turn facilitates its function of cellular senescence.

In conclusion our data reveal that resveratrol treatment induces premature senescence in human dermal fibroblasts that is mediated by DNA damage and by activation of p53-p21and Rb-p16 pathways. More importantly concomitant decline in the levels of SIRT1 and SIRT2 upon resveratrol treatment may be a cause for induction of senescence which is most likely mediated by a regulatory mechanism activated by DNA damage response. Based on the above data we put forward a possible model how resveratrol treatment induces senescence via down regulation of SIRT1 and SIRT2 shown in “Fig 11” (Fig 11).
